# Localized surface plasmon resonance sensing of hydrogen sulfide using zinc oxide film

**DOI:** 10.1038/s41598-025-12193-2

**Published:** 2025-08-01

**Authors:** Yuki Takimoto, Keiji Komatsu, Hiromasa Namiki, Kazuto Mochizuki, Tomoe Nakagawa, Kohki Nagata, Kazuki Komiya, Tomoko Gessei, Akira Monkawa

**Affiliations:** 1 Tokyo Metropolitan Industrial Technology Research Institute, 2-4-10 Aomi, Koto-ku, Tokyo, 135-0064 Japan; 2 Department of Materials Science and Bioengineering, Nagaoka University of Technology, 1603 Kamitomioka, Nagaoka, Niigata, 940-2188 Japan

**Keywords:** Localized surface plasmon resonance (LSPR), Au nanopattern, Zinc oxide, Hydrogen sulfide, Volatile sulfur compounds (VSCs), Periodontal disease, Imaging and sensing, Nanoparticles

## Abstract

A localized surface plasmon resonance (LSPR) sensor for hydrogen sulfide (H_2_S) has been developed by coating a gold nanopattern with zinc oxide (ZnO) film. H_2_S detection was achieved by monitoring changes in absorbance at a specific wavelength in the LSPR spectrum. The absorbance increased during exposure to H_2_S and remained constant after H_2_S ceased. These responses indicate that the sensor can measure the integrated concentrations of H_2_S. The sensitivity to H_2_S was enhanced with increasing relative humidity, enabling detection of 0.05–3 ppm H_2_S within 10 min at 70% relative humidity. The sensor’s sensitivity and LSPR spectrum could be restored by heating it at 500 °C in air. Notably, the sensor did not respond to methyl mercaptan or dimethyl sulfide, demonstrating high selectivity for H_2_S. This study highlights the potential of the ZnO-deposited LSPR sensor for highly sensitive and selective detection of H_2_S.

**Supplementary Information:**

The online version contains supplementary material available at 10.1038/s41598-025-12193-2.

## Introduction

Oral hygiene management is crucial for maintaining oral health, which contributes significantly to an individual’s quality of life (QOL) and healthy life expectancy^1^. However, oral diseases are widespread worldwide and greatly reduce the QOL of those affected^2^. For example, more than half of the global population suffers from periodontal disease, underscoring the growing importance of prevention. Many studies have reported that halitosis, or bad breath, is closely related with oral diseases such as periodontal disease^3–5^. Halitosis not only causes discomfort but also leads to social embarrassment^6^. Thus, halitosis testing is an effective oral hygiene technique for maintaining a good QOL. The primary causative agents of halitosis are volatile sulfur compounds (VSCs) produced by bacteria in the oral cavity^7–9^. The key VSCs include hydrogen sulfide (H_2_S), methyl mercaptan (CH_3_SH), and dimethyl sulfide ((CH_3_)_2_S). Studies have reported that the concentrations of H_2_S and CH_3_SH in oral diseases are typically at the sub-ppm order, with variations depending on the specific type of oral disease^10^. Therefore, monitoring VSCs with high sensitivity and selectivity is essential for effective oral hygiene management.

Conventional methods for measuring VSCs in clinical practice include organoleptic examination, electrochemical sensors, and gas chromatography (GC-MS). Organoleptic examination is a simple method in which a breath assessor rates halitosis on a scale of 0 to 5^11^. However, this method has several drawbacks, such as subjective evaluation, low reproducibility, the need for trained evaluators, and the risk of disease transmission. Electrochemical sensors can measure the concentration of H_2_S and CH_3_SH at the ppb level^3,6,12^. Although these sensors are easy to use, portable, and inexpensive, they cannot distinguish between the concentrations of different VSCs, and their readings can be affected by interference from other volatile organic compounds (VOCs) such as acetone and ethanol. Conventional GC-MS systems equipped with a flame photometric detector can measure three VSCs^13,14^. However, this technique requires expensive, large equipment and trained operators who must perform complicated procedures. A portable GC-MS has been developed to address these limitations^4,13^. This device can distinguish between the three VSCs with high sensitivity, is easy to handle, and is relatively inexpensive. Although clinical testing devices have become smaller and simpler, they are still too expensive and large for individuals to use for daily oral hygiene monitoring. Therefore, a smaller, less expensive sensor with high sensitivity and selectivity is needed.

In recent years, localized surface plasmon resonance (LSPR) has been applied to the production of useful substances by photocatalysis and the sensing of various materials such as proteins, VOCs, and inorganic gas molecules^15–21^. LSPR is the phenomenon in which conduction electrons on the surface of a metallic nanostructure collectively vibrate in resonance with light, resulting in absorption at a specific wavelength^22^. The absorption wavelength varies depending on the metal species (Au, Ag, Cu etc.), nanostructure, and the refractive index (RI) around the metal nanostructure. Target molecules are detected by changes in RI and the corresponding shift in the absorption wavelength induced by their approach to the metal nanostructures. To enhance sensitivity, metal nanostructures are often decorated with substances that interact with the target molecules. These nanostructures can be fabricated using cost-effective methods such as nanoimprint lithography and nanosphere lithography^23–26^. As a result, LSPR sensors are attracting attention as compact and inexpensive devices capable of detecting a wide range of substances with high sensitivity.

There are still few reports on LSPR sensors for detecting H_2_S. For example, Au nanoparticles dispersed in TiO_2_–NiO thin films have been reported to detect 2–10 ppm H_2_S at 350 °C via catalytic oxidation of H_2_S to SO_2_^27^. Another study demonstrated that a monolayer film of self-assembled Ag nanoparticles could detect 1–100 ppm H_2_S in N_2_ at 25 °C by monitoring changes in the LSPR absorption wavelength caused by Ag_2_S formation^28^. In our previous study, an Au nanopattern coated with ZIF-8, one of metal organic frameworks, was able to detect 0.1–20 ppm SO_2_ and 0.1–10 ppm H_2_S at 70% relative humidity (RH) and 25°C^29^. Despite the relatively high sensitivity of this sensor, ZIF-8 degraded over time when exposed to H_2_S and SO_2_, leading to reduced sensitivity. Furthermore, the sensor also responded to changes in RH due to water absorption by the porous nature of ZIF-8.

Zinc oxide (ZnO) has been widely used as a versatile functional material, serving both as a sensing material for various gases, including H_2_S, and as an adsorbent for the desulfurization of factory exhaust gases^30–33^. Semiconductor sensors employing ZnO have been shown to detect H_2_S in the sub-ppm to ppm range at operating temperatures between room temperature and 300°C^34,35^. ZnO reacts with H_2_S to form ZnS, a reactivity that has been leveraged in surface plasmon resonance (SPR) sensors. These sensors, using optical fibers, have been developed for the detection of 10–100 ppm H_2_S in N_2_ at room temperature^31,36–38^. These sensors were fabricated by coating ZnO and Cu or Ag layers onto the unclad portions of optical fibers. The characteristics of ZnO make it a promising candidate for application in LSPR sensors. Unlike porous materials, non-porous ZnO does not absorb water, even under high humidity conditions, which minimizes water-induced responses. Additionally, ZnO-coated LSPR sensors can be regenerated by heating after H_2_S measurement, as ZnS can be oxidized back to ZnO in air at 500 °C or higher^39^. Therefore, in this study, LSPR sensors for H_2_S detection were developed by depositing ZnO onto the surface of Au nanopatterns. The effects of RH on sensor sensitivity, the reusability of the sensors following heating, and their selectivity for H_2_S over CH_3_SH and (CH_3_)_2_S were thoroughly investigated.

## Results and discussion

### Characteristics of ZnO-deposited LSPR sensors and their response to H_2_S

**Fig. 1:**
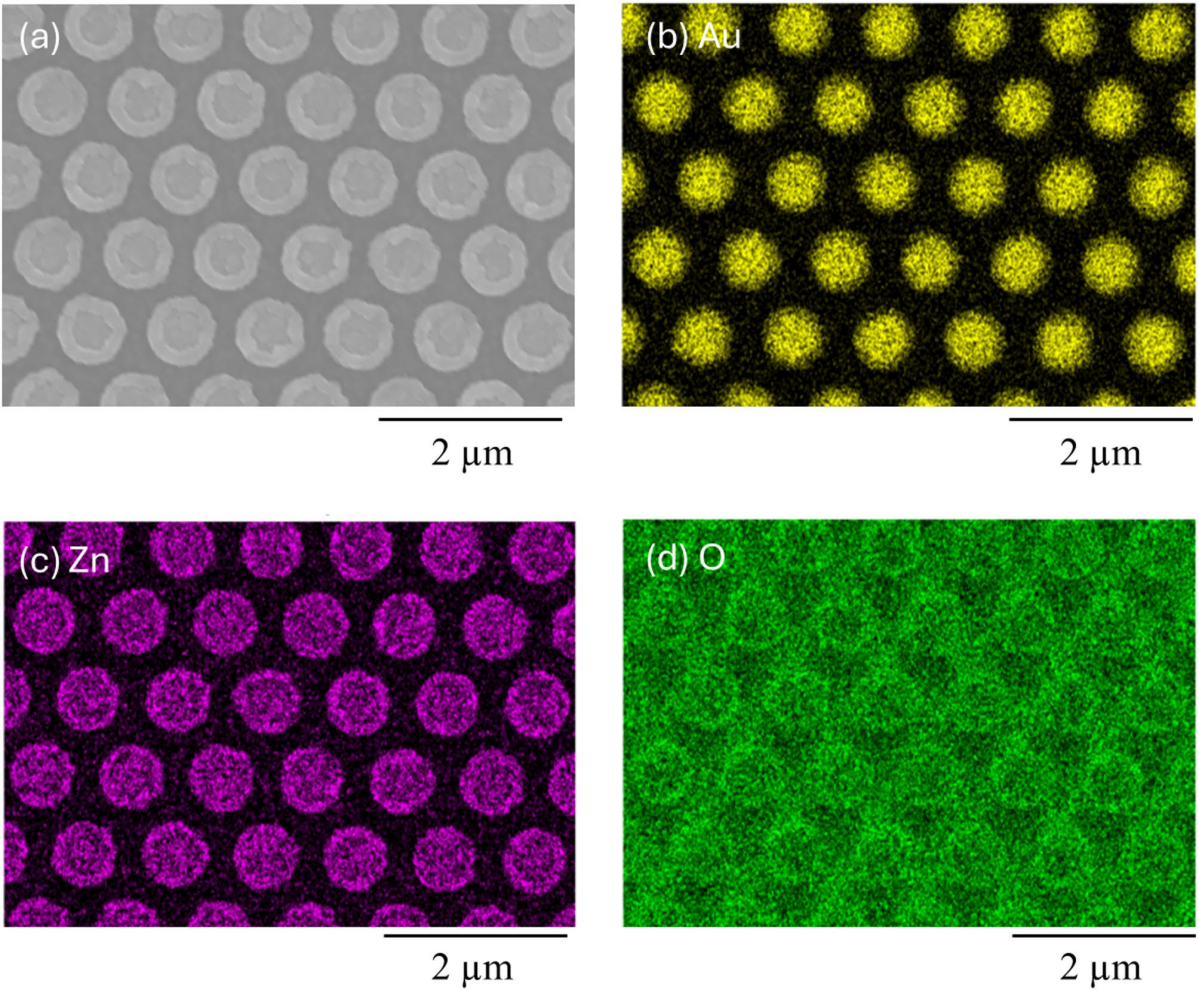
(**a**) SEM image of a ZnO-deposited Au nanopattern on a SiO_2_ glass substrate. SEM-EDS elemental mapping showing the (**b**) Au, (**c**) Zn and (**d**) O distribution.

**Fig. 2:**
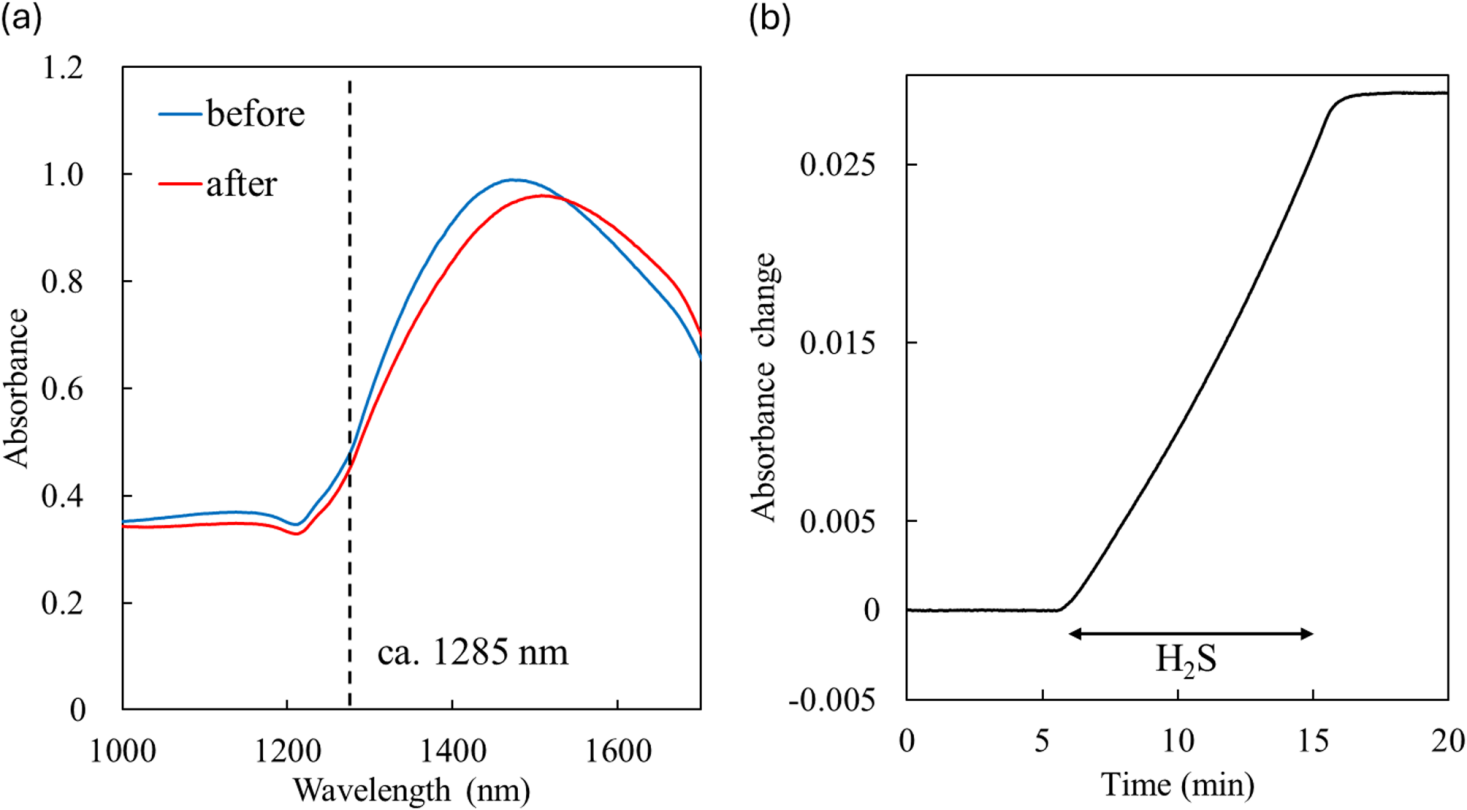
(**a**) Localized surface plasmon resonance (LSPR) spectra of a ZnO-deposited sensor before (blue line) and after exposure to 3 ppm H_2_S gas at 70% RH for 10 min (red line). The black dashed line indicates the position of the inflection point where the absorbance change was measured. The wavelength in this spectrum was approximately 1285 nm. (**b**) Absorbance changes of the ZnO-deposited sensor induced by 3 ppm H_2_S from 5 to 15 min at 70% RH.

 ZnO-deposited LSPR sensors were fabricated by chemical vapor deposition of a 30 nm thick ZnO layer onto an Au nanopattern with a diameter of 650 nm, a pitch of 1000 nm, and a height of 120 nm, on a SiO_2_ glass substrate (Fig. 1 and S1). The thickness of the ZnO layer was estimated based on the deposition rate. ZnO uniformly and thinly coated the Au nanopattern, as shown in Fig. 1. The refraction peaks of ZnO and Au were observed by X-ray diffraction measurement (Fig. S2). Our previous simulation results revealed that the sensitivity of the Au nanopattern with the LSPR absorbance spectrum in the near-infrared (NIR) region was higher than in the visible region^40^. In addition, Au is more durable to the corrosive H_2_S than Ag or Cu. Therefore, the Au nanopattern showing LSPR in the NIR region was also used in this study for the highly sensitive detection of H_2_S (Fig. 2a).

The LSPR absorbance spectra of the sensors exhibited a shift to higher wavelengths upon exposure to H_2_S gas. For instance, Fig. 2a shows the LSPR spectra of a sensor before and after exposure to 3 ppm H_2_S for 10 min at 70% RH. This shift indicates an increase in the RI surrounding the Au nanopattern, changing from ZnO (*n* = 1.57 at 1285 nm) to ZnS (*n* = 2.28 at 1285 nm)^41^. Approximately 30 sensors were used to assess sensor performance. The LSPR spectra of these sensors varied slightly; the average peak wavelength and absorbance were 1472 ± 18 nm and 1.10 ± 0.17, respectively. Wavelengths associated with maximum absorbance changes due to H_2_S exposure also varied depending on spectral differences and H_2_S concentration. To standardize measurements, absorbance changes were measured at the inflection point of each sensor’s spectrum before exposure, rather than at the wavelength showing the largest absorbance change. This approach facilitates the use of portable, low-cost sensors, as absorbance can be measured using a photodiode and band-pass filter instead of a more expensive spectrometer.

Figure 2b illustrates the absorbance change of a sensor induced by 3 ppm H_2_S exposure for 10 min at 70% RH, at approximately 1285 nm. For clarity, decreases in absorbance caused by H_2_S exposure were treated as positive values. The absorbance increased almost linearly during H_2_S exposure as ZnO reacted with H_2_S to form ZnS. Since ZnS does not oxidize back to ZnO at room temperature, the absorbance remained stable even after H_2_S exposure ceased. Consequently, the sensor can provide integrated H_2_S concentrations, similar to gas detector tubes.

### Effect of RH on sensitivity

 The absorbance remained relatively unchanged across RH levels from < 1–70% in the absence of H_2_S (Fig. S3). Minor variations in absorbance could be attributed to water adsorption on the ZnO film surface. By contrast, LSPR sensors coated with the porous material (ZIF-8) in our previous study exhibited significant RH sensitivity, with absorbance changes comparable to those induced by 10 ppm H_2_S at 70% RH^29^. These results highlight the non-porous nature of ZnO films which has the benefit of suppressing absorbance changes due to H_2_O itself. Exhaled breath typically contains RH levels ranging from 42 to 91%, making the films’ nature advantageous for practical H_2_S measurements in moist conditions^42^.

**Fig. 3:**
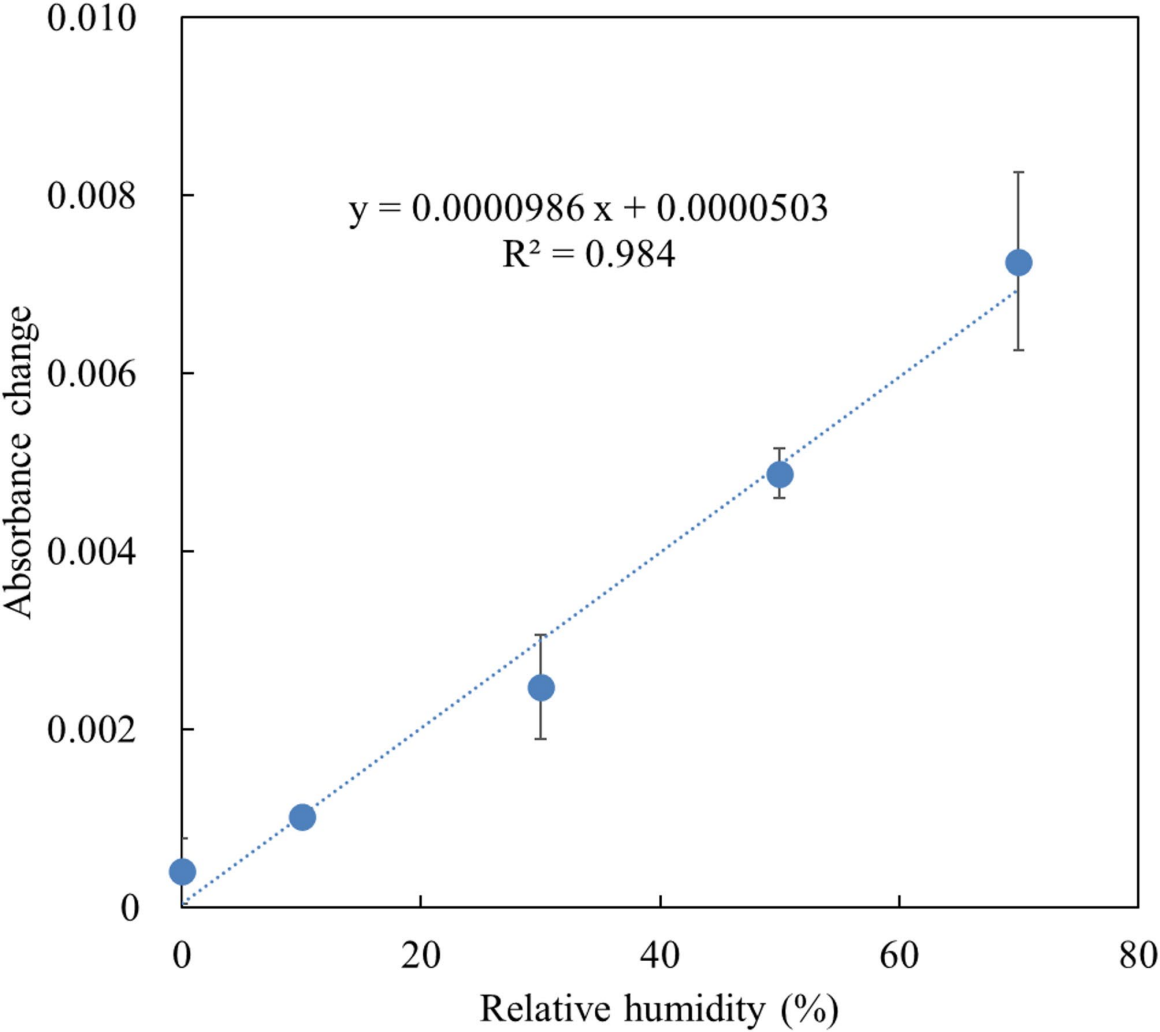
Absorbance changes induced by 1 ppm H_2_S exposure versus relative humidity (*n* = 3).

Figure 3 shows the absorbance changes induced by 1 ppm H_2_S at RH levels from < 1–70%. The absorbance increased with RH and was approximately 18 times greater at 70% RH compared to < 1% RH. This observation indicates that water enhanced the reaction between ZnO and H_2_S. This result is consistent with Zhao et al.’s report on the increased H_2_S capacity of ZnO adsorbents in the presence of water^43^. The proposed mechanism is as follows: hydroxyl groups derived from H_2_O on the ZnO surface modify the reaction process and lower the reaction activation energy, thereby enhancing ZnO activity. On the other hand, at very high humidity, the activity of ZnO is adversely reduced by H_2_O^43^. It is possible that the sensitivity of the sensors may decrease at humidities above 70% RH. The sensitivity above 70% RH could not be verified because of the limitation of this experimental system. The high sensitivity under high humidity (at least 70% RH) is also advantageous for practical measurements, although it is necessary to account for the effect of RH on absorbance changes.

### Regeneration of the sensors

**Fig. 4:**
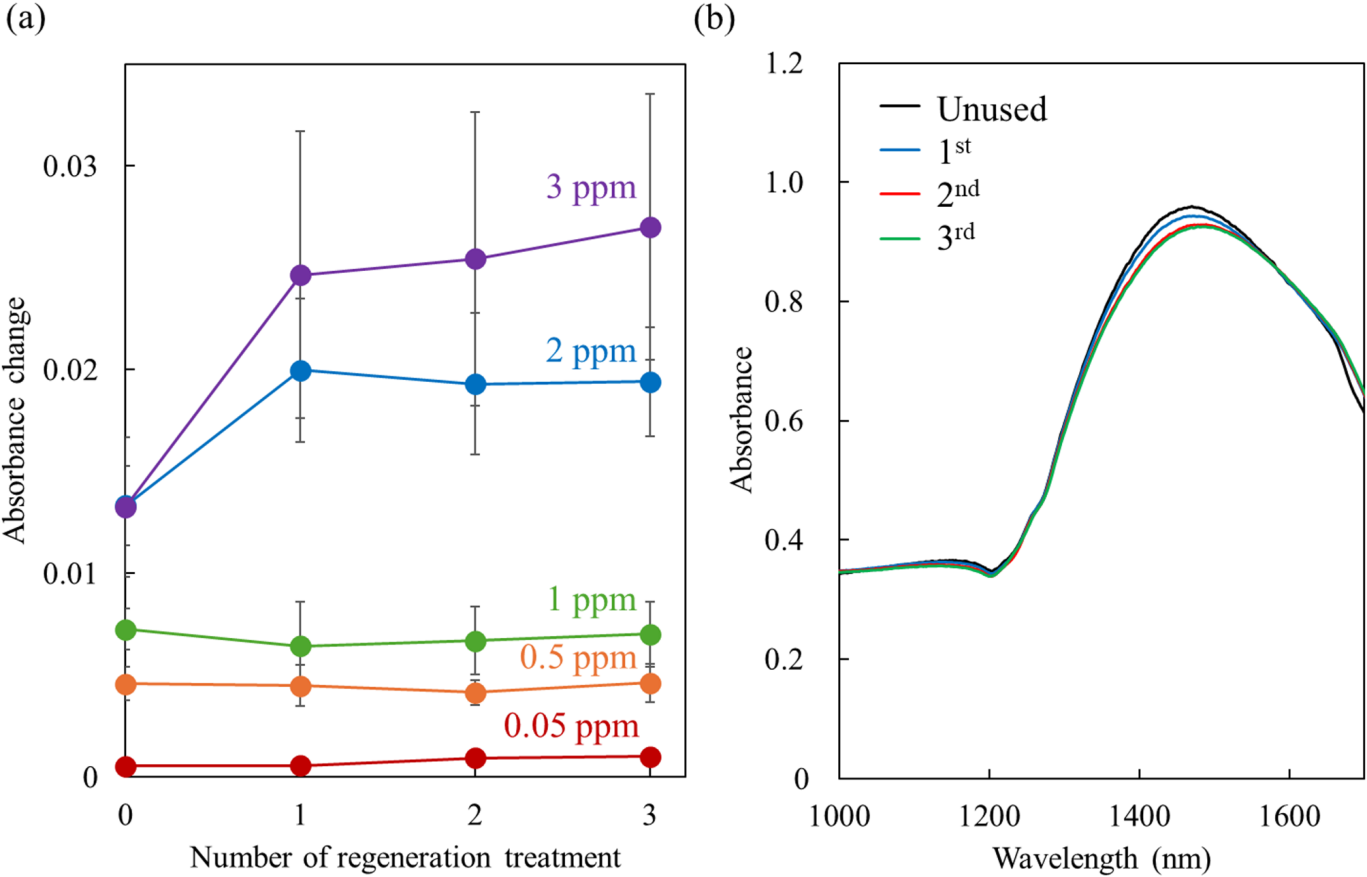
(**a**) Absorbance changes induced by 0.05, 0.5, 1, 2, and 3 ppm H_2_S exposure for 10 min after each regeneration treatment (heat at 500 °C in air for 1 h after H_2_S exposure) (*n* = 3). At the initial regeneration the sensors were not heated and were used for the first time. (**b**) LSPR spectra of a ZnO-deposited sensor unused (black line), after the 1 st (blue line), the 2nd (red line), and the 3rd regeneration treatment (green line).

 ZnS is oxidized to ZnO when heated above 500 °C in air^39^. Therefore, after exposure to H_2_S, the ZnO-deposited sensors were heated at 500 °C in air for 1 h to regenerate them. The absorbance changes remained nearly constant from the first to the third regeneration cycles (Fig. 4a). This indicates that the sensor can be reused multiple times through a heat regeneration process. The large error bars were attributed to variations in the sensitivity of individual sensors, which could be improved by ensuring consistent sensor quality. In addition, the deviation became larger because the absorbance changed more significantly with a higher concentration of H_2_S. For the initial regeneration (i.e., the first use of the sensors), the absorbance changes for 2 and 3 ppm H_2_S were smaller than those after the first to third regenerations. This may be due to the reaction of ZnO with 2–3 ppm H_2_S and the oxidation of ZnS, which could cause deformation or cracking of the ZnO film, increasing its surface area and enhancing reactivity. However, no significant difference in the film’s morphology was observed.

After the first two regenerations, the LSPR spectra showed reduced absorbance and a 10 nm peak shift (Fig. 4b). This result suggests deformation of the ZnO film and/or partial integration of unoxidized ZnS. By the third regeneration, the spectra stabilized and were nearly identical to those from the second regeneration, indicating that the film reached a steady state and that the sensors could be reused reliably.

### Calibration curve at 70% RH

**Fig. 5:**
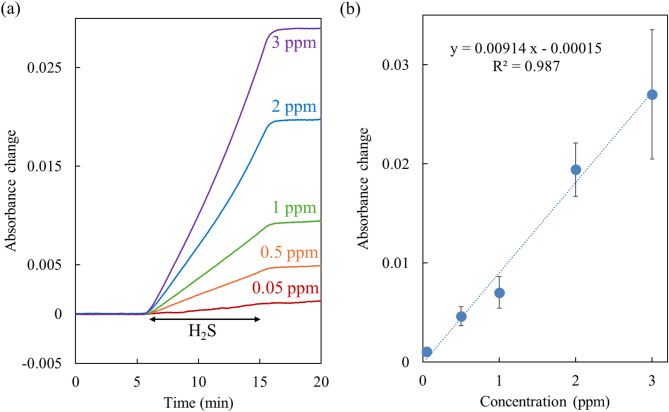
(**a**) Absorbance changes induced by 0.05, 0.5, 1, 2, and 3 ppm H_2_S from 5 to 15 min at 70% RH. (**b**) Absorbance changes versus the concentrations of H_2_S at 70% RH after the third regeneration treatment (*n* = 3).

 The responses to 0.05–2 ppm H_2_S at 70% RH after the third regeneration followed a comparable trend of 3 ppm H_2_S (Fig. 5a). Figure 5b shows the linear calibration curve for H_2_S concentrations ranging from 0.05 ppm to 3 ppm at 70% RH after the third regeneration. The sensitivity obtained from the slope of the calibration curve was 0.00914 ppm^−1^. The limit of detection (LOD, 3σ) was 0.02 ppm, comparable to that of commercial electrochemical sensors used in clinical practice. The LOD was below the odor recognition threshold for H_2_S (0.02–0.13 ppm), enabling the sensor to detect H_2_S in exhaled breath at concentrations reported for periodontal disease patients (~ 0.1 ppm)^10,44^. Longer exposure times could allow detection of even lower concentrations as the sensor obtains integrated concentration. Furthermore, the sensor’s applicability extends beyond oral hygiene, making it suitable for monitoring H_2_S emissions from industrial and natural sources such as factories and volcanoes^45^.

### Selectivity to H_2_S

**Fig. 6:**
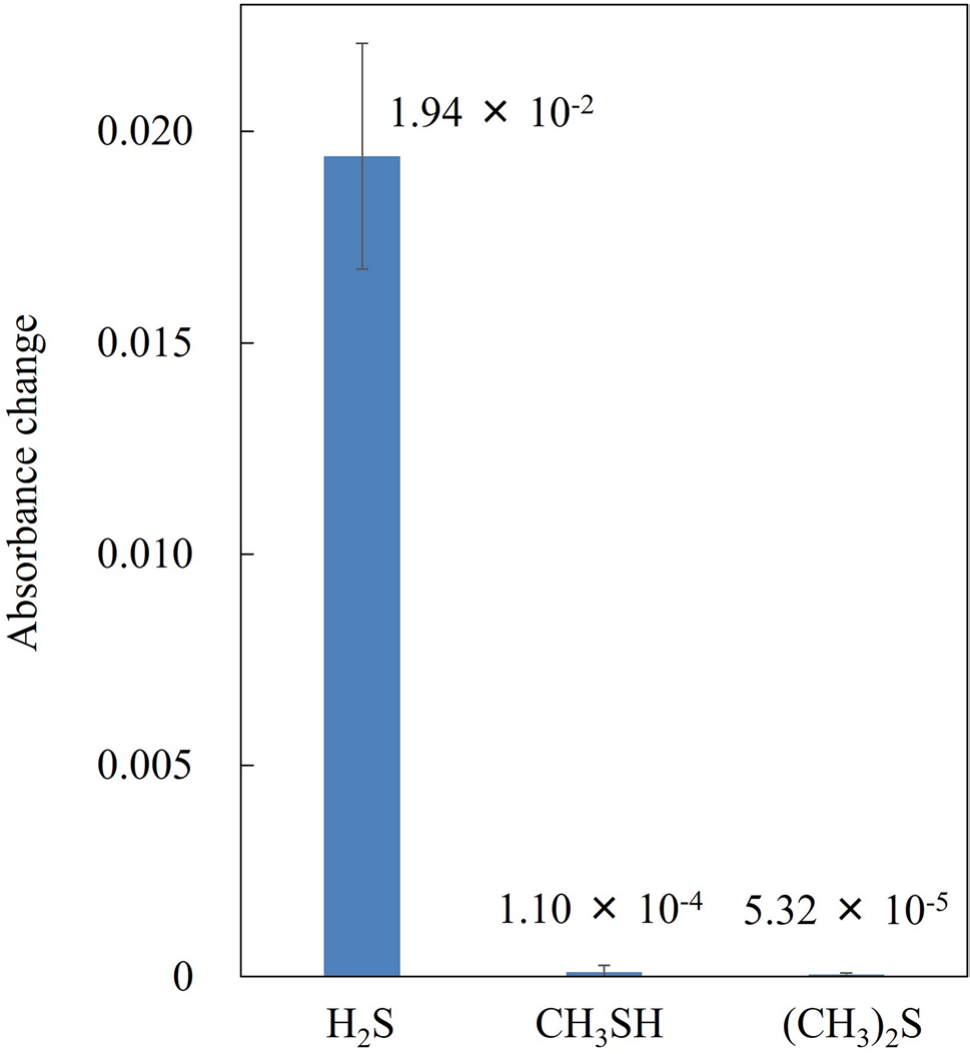
Absorbance changes induced by 10-min exposure to 2 ppm H_2_S, CH_3_SH, and (CH_3_)_2_S at 70% RH.

 The sensors were exposed to 2 ppm of CH_3_SH and (CH_3_)_2_S, representative VSCs present in exhalation alongside H_2_S, at 70% RH and were barely detected, as shown in Fig. 6. The absorbance change for H_2_S was approximately 177 and 365 times larger than those for CH_3_SH and (CH_3_)_2_S, respectively, demonstrating the high selectivity of the sensors for H_2_S. Although CH_3_SH and (CH_3_)_2_S can adsorb onto the surface of ZnO with Zn-S bonding, the adsorption amounts appeared too small to cause significant changes in the RI around the Au nanopattern^46,47^. Similarly, these sensors are unlikely to respond to other VOCs that neither react with nor adsorb significantly onto ZnO. On the other hand, LSPR sensors coated with mesoporous silica, as developed in our previous study, could detect a variety of VOCs due to the non-selective absorption capability of porous materials^48^. By contrast, the ZnO film lacks such absorption capabilities due to its non-porous nature. This selective chemical reactivity and absence of absorption in ZnO films contributes to its high selectivity for H_2_S. Therefore, the ZnO-deposited sensor demonstrates excellent potential for selectively detecting H_2_S in exhaled air containing various VOCs. In the future, LSPR sensors for CH_3_SH and (CH_3_)_2_S could be developed by fabricating selective films that specifically react with these compounds. Highly selective VSC sensors would be invaluable for diagnosing and identifying various oral diseases.

## Conclusions

An LSPR sensor for highly sensitive and selective detection of H_2_S was developed by depositing ZnO onto an Au nanopattern on a glass substrate. The sensor exhibited a linear increase in absorbance during H_2_S exposure, which remained stable after exposure ceased, enabling the measurement of the integrated H_2_S concentration. Sensitivity was enhanced with increasing humidity; at 70% RH, the sensor detected 0.05–3 ppm H_2_S within 10 min. In contrast, CH_3_SH and (CH_3_)_2_S were not detected, underscoring the sensor’s high selectivity. The sensitivity was nearly fully recovered after H_2_S exposure by heating the sensors at 500 °C in air, allowing repeated use for at least three cycles. Thus, the developed sensor shows strong potential for applications in monitoring H_2_S in exhaled breath and other settings. In the future, applications for measuring other gases such as SO_2_ will be investigated because the sensor can have the ability to detect gases that react with ZnO.

## Methods

### Fabrication of a ZnO-deposited LSPR sensor

 An Au nanopattern was fabricated on a SiO_2_ glass substrate (10 mm × 10 mm × 1 mm) in collaboration with Kyodo International, Inc (Kanagawa, Japan). The metal coating process was conducted using a sputtering machine (EIS-230; Elionix Inc., Tokyo, Japan), with a 20 nm Si adhesion layer between the Au nanopattern and the SiO_2_ glass substrate. Additional processes, such as nanoimprinting, were also carried out by Kyodo International, Inc. After the substrate was heated at 450 °C for 1 h, a 30 nm thick ZnO layer was deposited onto the Au nanopattern via chemical vapor deposition. The reactant, bis(dipivaloylmethanato)zinc (Zn(DPM)_2_, Kojundo Chemical Laboratory Co., Ltd., Saitama, Japan), was vaporized using an electric heater. The reactant vapor was carried by nitrogen gas flowing at a rate of 1.5 L/min and sprayed directly onto the substrates mounted on an electric heater. The distance between the nozzle and the substrate was kept at 25 mm. Vaporizer (Tv) and substrate (Ts) temperatures were measured using K-type thermocouples and maintained at 120 and 400 ^o^C, respectively. Surface topography was analyzed using an atomic force microscope (AFM, SPM-9700HT; Shimadzu Corp., Kyoto, Japan) and a field emission scanning electron microscope (FE-SEM, SU-8600; Hitachi High-Tech Corp., Tokyo, Japan). The X-ray diffraction pattern was obtained with an X-ray diffractometer (SmartLab SE; Rigaku Corp., Tokyo, Japan).

### Gas detection experiments

 The gas-sensing system used for this study was described in detail in our previous work^29^. Air containing 0.05–3 ppm H_2_S, 2 ppm CH_3_SH, or 2 ppm (CH_3_)_2_S was generated using the permeation-tube method. During the 20-min measurement period, background air flowed continuously, with air containing the target gas introduced from 5 to 15 min at a controlled flow rate of 500 mL/min (MQV 9500; Azbil Corp., Tokyo, Japan). The exposure time to the target gas (10 min) was chosen to detect 0.05 ppm H_2_S at 70% RH. The RH of the air was controlled from < 1–70% by mixing dry air (RH < 1%) with saturated water vapor. 70% RH was the maximum value in this system. LSPR absorbance spectra were measured using a tungsten halogen lamp (HL-2000; Ocean Optics Inc., Largo, FL, USA) and a near-infrared spectrometer (NIRQuest; Ocean Optics Inc.) connected to the sensing chamber via optical fibers. Sensor responses were recorded as absorbance changes at a fixed wavelength, determined from the inflection point of the pre-exposure LSPR spectrum. The experiments were conducted at room temperature (25 °C). After exposure to air containing H_2_S, sensors were regenerated by heating at 500 °C in air for 1 h to oxidize ZnS back to ZnO.

## Supplementary Information

Below is the link to the electronic supplementary material.

Supplementary material 1 (PDF 289.1 kb)

## Data Availability

All data of this study are available from the corresponding author on reasonable request.

## References

[1] Patel, J. et al. Oral health for healthy ageing. *Lancet Healthy Longev.* **2**, e521–e527. 10.1016/S2666-7568(21)00142-2 (2021). 36098001 10.1016/S2666-7568(21)00142-2

[2] Duangthip, D. & Chu, C. H. Challenges in oral hygiene and oral health policy. *Front. Oral Health*. **1**, 575428. 10.3389/froh.2020.575428 (2020). 35047981 10.3389/froh.2020.575428 PMC8757757

[3] Yaegaki, K., Coil, J. M. & Examination Classification, and treatment of halitosis; clinical perspectives. *J. Can. Dent. Assoc.* **66**, 257–261 (2000). https://www.cda-adc.ca/jcda/vol-66/issue-5/257.html 10833869

[4] Nakhleh, M. K., Quatredeniers, M. & Haick, H. Detection of halitosis in breath: Between the past, present, and future. *Oral Dis.* **24**, 685–695. 10.1111/odi.12699 (2018). 28622437 10.1111/odi.12699

[5] Wu, D. D. et al. Role of hydrogen sulfide in oral disease. *Oxid. Med. Cell. Longev.* **2022**, 1886277. 10.1155/2022/1886277 (2022). 35116090 10.1155/2022/1886277 PMC8807043

[6] Wu, J., Cannon, R. D., Ji, P., Farella, M. & Mei, L. Halitosis: prevalence, risk factors, sources, measurement and treatment - a review of the literature. *Aust Dent. J.* **65**, 4–11. 10.1111/adj.12725 (2020). 31610030 10.1111/adj.12725

[7] Bollen, C. M. & Beikler, T. Halitosis: The multidisciplinary approach. *Int. J. Oral Sci.* **4**, 55–63. 10.1038/ijos.2012.39 (2012). 22722640 10.1038/ijos.2012.39 PMC3412664

[8] Cortelli, J. R., Barbosa, M. D. S. & Westphal, M. A. Halitosis: A review of associated factors and therapeutic approach. *Braz. Oral Res.* 44–54. 10.1590/S1806-83242008000500007 (2008). 19838550 10.1590/s1806-83242008000500007

[9] Aylikci, B. U., Colak, H. & Halitosis From diagnosis to management. *J. Nat. Sci. Biol. Med.* **4**, 14–23. 10.4103/0976-9668.107255 (2013). 23633830 10.4103/0976-9668.107255 PMC3633265

[10] Lee, Y. H., Shin, S. I. & Hong, J. Y. Investigation of volatile sulfur compound level and halitosis in patients with gingivitis and periodontitis. *Sci. Rep.* **13**, 13175. 10.1038/s41598-023-40391-3 (2023). 37580412 10.1038/s41598-023-40391-3 PMC10425441

[11] Aydin, M. Criticism of the organoleptic examination for the diagnosis of oral halitosis. *J. Breath. Res.* **17**10.1088/1752-7163/ac8faf (2022). 10.1088/1752-7163/ac8faf 36067739

[12] Patil, S. H., Kulloli, A. & Kella, M. Unmasking oral malodor: A review. *People’s J. Sci. Res.* **5**, 61–67. 10.5281/zenodo.8267370 (2012).

[13] Murata, T. et al. Development of a compact and simple gas chromatography for oral malodor measurement. *J. Periodontol.* **77**, 1142–1147. 10.1902/jop.2006.050388 (2006). 16805675 10.1902/jop.2006.050388

[14] Yoneda, M., Suzuki, N. & Hirofuji, T. Current status of the techniques used for halitosis analysis. *Austin Chromatogr.* **2** (2015).

[15] Song, H. et al. Light-enhanced carbon dioxide activation and conversion by effective plasmonic coupling effect of Pt and Au nanoparticles. *ACS Appl. Mater. Interfaces* **10**, 408–416. 10.1021/acsami.7b13043 (2018). 29226665 10.1021/acsami.7b13043

[16] Luo, S., Ren, X., Lin, H., Song, H. & Ye, J. Plasmonic photothermal catalysis for solar-to-fuel conversion: Current status and prospects. *Chem. Sci.* **12**, 5701–5719. 10.1039/D1SC00064K (2021). 34168800 10.1039/d1sc00064k PMC8179669

[17] Huang, H. et al. Near-Infrared Plasmon-Driven nitrogen photofixation achieved by assembling Size-Controllable gold nanoparticles on TiO_2_ nanocavity arrays. *ACS Sustain. Chem. Eng.* **11**, 10993–11001. 10.1021/acssuschemeng.2c07086 (2023).

[18] Chen, K. J. & Lu, C. J. A vapor sensor array using multiple localized surface plasmon resonance bands in a single UV-vis spectrum. *Talanta* **81**, 1670–1675. 10.1016/j.talanta.2010.03.023 (2010). 20441956 10.1016/j.talanta.2010.03.023

[19] Kreno, L. E., Hupp, J. T. & Van Duyne, R. P. Metal–organic framework thin film for enhanced localized surface plasmon resonance gas sensing. *Anal. Chem.* **82**, 8042–8046. 10.1021/ac102127p (2010). 20839787 10.1021/ac102127p

[20] Cao, J., Sun, T. & Grattan, K. T. V. Gold nanorod-based localized surface plasmon resonance biosensors: A review. *Sens. Actuators, B Chem.* **195**, 332–351. 10.1016/j.snb.2014.01.056 (2014).

[21] Jo, N. R., Lee, K. J. & Shin, Y. B. Enzyme-coupled nanoplasmonic biosensing of cancer markers in human serum. *Biosens. Bioelectron.* **81**, 324–333. 10.1016/j.bios.2016.03.009 (2016). 26985585 10.1016/j.bios.2016.03.009

[22] Willets, K. A. & Van Duyne, R. P. Localized surface plasmon resonance spectroscopy and sensing. *Annu. Rev. Phys. Chem.* **58**, 267–297. 10.1146/annurev.physchem.58.032806.104607 (2007). 17067281 10.1146/annurev.physchem.58.032806.104607

[23] Haes, A. J. et al. Plasmonic materials for surface-enhanced sensing and spectroscopy. *MRS Bull.* **30**, 368–375. 10.1557/mrs2005.100 (2005).

[24] Yu, C. C. & Chen, H. L. Nanoimprint technology for patterning functional materials and its applications. *Microelectron. Eng.* **132**, 98–119. 10.1016/j.mee.2014.10.015 (2015).

[25] Choi, M. et al. Fabrication and characterization of gold nanocrown arrays on a gold film for a high-sensitivity surface plasmon resonance biosensor. *Thin Solid Films* **587**, 43–46. 10.1016/j.tsf.2014.11.047 (2015).

[26] Kang, M., Losego, M., Sachet, E., Maria, J. P. & Franzen, S. Near-infrared optical extinction of indium tin oxide structures prepared by nanosphere lithography. *ACS Photonics* **3**, 1993–1999. 10.1021/acsphotonics.6b00649 (2016).

[27] Della Gaspera, E. et al. Au nanoparticles in nanocrystalline TiO_2_ – NiO films for SPR-based, selective H_2_S gas sensing. *Chem. Mater.* **22**, 3407–3417. 10.1021/cm100297q (2010).

[28] Chen, R., Morris, H. R. & Whitmore, P. M. Fast detection of hydrogen sulfide gas in the ppmv range with silver nanoparticle films at ambient conditions. *Sens. Actuators, B Chem.* **186**, 431–438. 10.1016/j.snb.2013.05.075 (2013).

[29] Takimoto, Y. et al. Localized surface plasmon resonance sensing of SO_2_ and H_2_S using zeolitic imidazolate framework-8. *Sens. Actuators B: Chem.* **383**, 133585. 10.1016/j.snb.2023.133585 (2023).

[30] Özgür, Ü., Hofstetter, D. & Morkoç, H. ZnO devices and applications: A review of current status and future prospects. *Proc. IEEE.* **98**, 1255–1268. 10.1109/jproc.2010.2044550 (2010).

[31] Shah, M. S., Tsapatsis, M. & Siepmann, J. I. Hydrogen sulfide capture: From absorption in polar liquids to oxide, zeolite, and metal-organic framework adsorbents and membranes. *Chem. Rev.* **117**, 9755–9803. 10.1021/acs.chemrev.7b00095 (2017). 28678483 10.1021/acs.chemrev.7b00095

[32] Georgiadis, A., Charisiou, N. & Goula, M. Removal of hydrogen sulfide from various industrial gases: A review of the most promising adsorbing materials. *Catalysts* **10**, 521. 10.3390/catal10050521 (2020).

[33] Komatsu, K., Iwamoto, T., Ito, H. & Saitoh, H. THz gas sensing using terahertz time-domain spectroscopy with ceramic architecture. *ACS Omega* **7**, 30768–30772. 10.1021/acsomega.2c01635 (2022). 36092607 10.1021/acsomega.2c01635 PMC9453963

[34] Hosseini, Z. S., Mortezaali, A., Iraji zad, A. & Fardindoost, S. Sensitive and selective room temperature H_2_S gas sensor based on Au sensitized vertical ZnO nanorods with flower-like structures. *J. Alloys Compd.* **628**, 222–229. 10.1016/j.jallcom.2014.12.163 (2015).

[35] Chen, Y., Xu, P., Xu, T., Zheng, D. & Li, X. *ZnO*-nanowire size effect induced ultra-high sensing response to ppb-level H_2_S. *Sens. Actuators, B Chem.* **240**, 264–272. 10.1016/j.snb.2016.08.120 (2017).

[36] Wiheeb, A. D. et al. Present technologies for hydrogen sulfide removal from gaseous mixtures. *Rev. Chem. Eng.* **29**. 10.1515/revce-2013-0017 (2013).

[37] Tabassum, R., Mishra, S. K. & Gupta, B. D. Surface plasmon resonance-based fiber optic hydrogen sulphide gas sensor utilizing Cu-ZnO thin films. *Phys. Chem. Chem. Phys.* **15**, 11868–11874. 10.1039/c3cp51525g (2013). 23764905 10.1039/c3cp51525g

[38] Usha, S. P., Mishra, S. K. & Gupta, B. D. Fiber optic hydrogen sulfide gas sensors utilizing ZnO thin film/zno nanoparticles: A comparison of surface plasmon resonance and lossy mode resonance. *Sens. Actuators B: Chem.* **218**, 196–204. 10.1016/j.snb.2015.04.108 (2015).

[39] Hong, E. & Kim, J. H. Oxide content optimized ZnS–ZnO heterostructures via facile thermal treatment process for enhanced photocatalytic hydrogen production. *Int. J. Hydrogen Energy ***39**, 9985–9993. 10.1016/j.ijhydene.2014.04.137 (2014).

[40] Takimoto, Y. et al. Detection of SO_2_ at the ppm level with localized surface plasmon resonance (LSPR) sensing. *Plasmonics* **15**, 805–811. 10.1007/s11468-019-01099-1 (2019).

[41] Polyanskiy, M. N. Refractive index database. https://refractiveindex.info. Accessed on 2024-10-11.

[42] Mansour, E. et al. Measurement of temperature and relative humidity in exhaled breath. *Sens. Actuators B: Chem.* **304**, 127371. 10.1016/j.snb.2019.127371 (2020).

[43] Zhao, Y. et al. Critical role of water on the surface of ZnO in H_2_S removal at room temperature. *Ind. Eng. Chem. Res.* **57**, 15366–15374. 10.1021/acs.iecr.8b03431 (2018).

[44] Costigan, M. G. Hydrogen sulfide: UK occupational exposure limits. *Occup. Environ. Med.* **60**, 308–312. 10.1136/oem.60.4.308 (2003). 12660382 10.1136/oem.60.4.308 PMC1740516

[45] Batterman, S., Grant-Alfieri, A. & Seo, S. H. Low level exposure to hydrogen sulfide: A review of emissions, community exposure, health effects, and exposure guidelines. *Crit. Rev. Toxicol. ***53**, 244–295. 10.1080/10408444.2023.2229925 (2023). 37431804 10.1080/10408444.2023.2229925 PMC10395451

[46] Casarin, M. et al. An experimental and theoretical study of the interaction of CH_3_OH and CH_3_SH with ZnO. *J. Chem. SOC. Faraday Trans.* **92**, 3247–3258. 10.1039/ft9969203247 (1996).

[47] Kim, S., Somaratne, R. M. D. S. & Whitten, J. E. Effect of adsorption on the photoluminescence of zinc oxide nanoparticles. *J. Phys. Chem. C*. **122**, 18982–18994. 10.1021/acs.jpcc.8b04715 (2018).

[48] Monkawa, A. et al. With high sensitivity and with wide-dynamic-range localized surface-plasmon resonance sensor for volatile organic compounds. *Sens. Actuators B: Chem.* **196**, 1–9. 10.1016/j.snb.2014.01.066 (2014).

