# Supplementary material for: Localized surface plasmon resonance sensing of hydrogen sulfide using zinc oxide film
Source: Sci Rep. 2025 Aug 1;15:28087. doi: 10.1038/s41598-025-12193-2 (PMC12317021; doi:10.1038/s41598-025-12193-2)
Supplement: Supplementary file 1 — Supplementary material 1 (PDF 289.1 kb) [file 41598_2025_12193_MOESM1_ESM.pdf]

Supplementary Information

Localized Surface Plasmon Resonance Sensing of Hydrogen Sulfide using Zinc Oxide Film

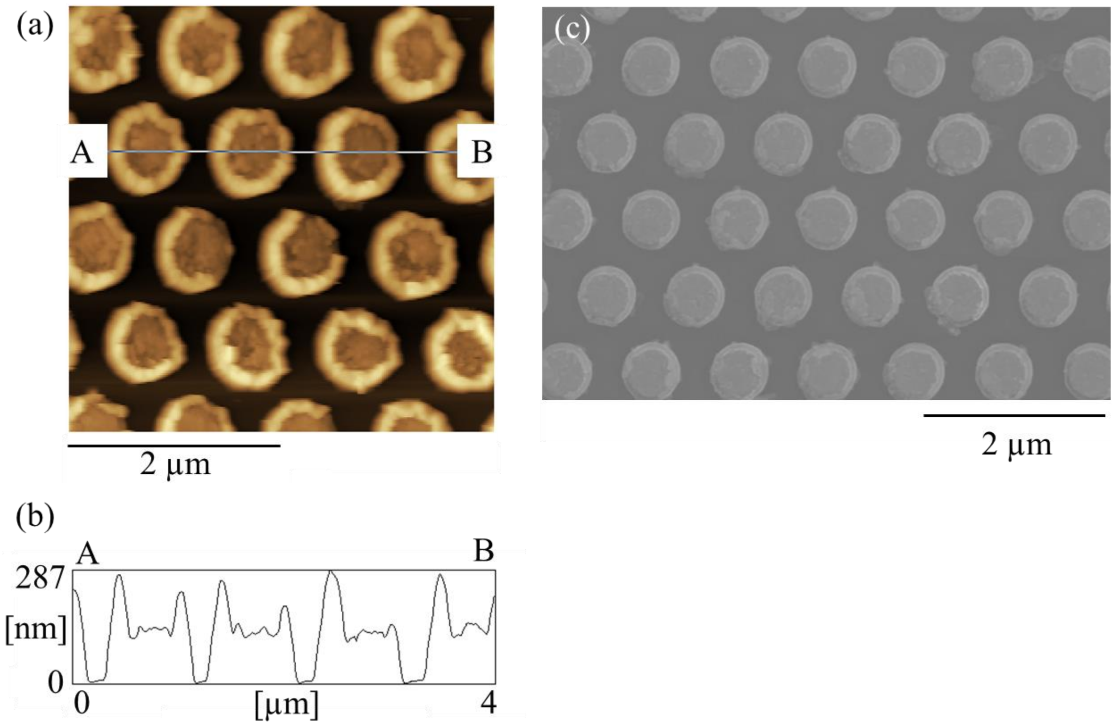

Fig. S1. (a) AFM image, (b) cross-sectional profile and SEM image of an Au nanopattern.

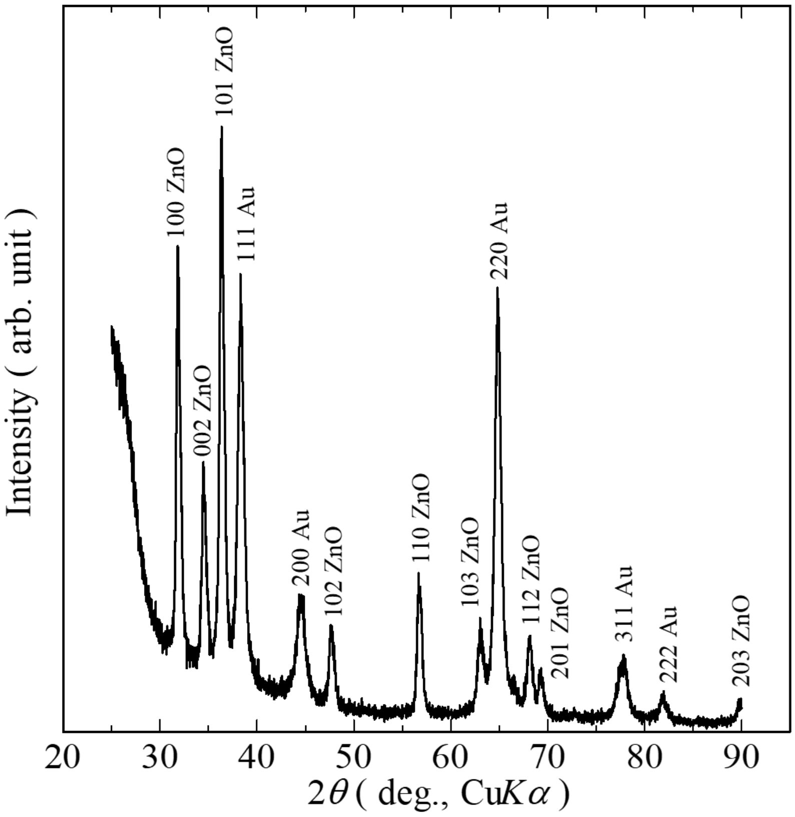

Fig. S2. X-ray diffraction pattern of a ZnO-deposited Au nanopattern.

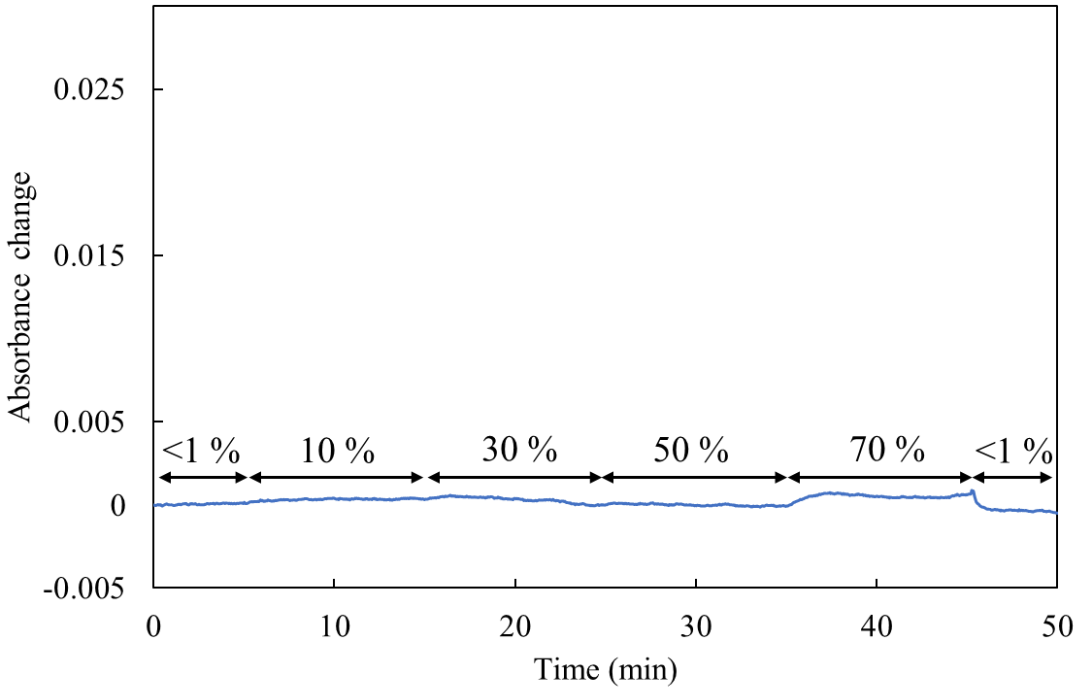

Fig. S3. Absorbance change of the ZnO-deposited sensor induced by varying RH from <1 % to 70 % for every 10 min without H_2S.
